# Multidisciplinary approaches to identifying and managing global airways disease: Expert recommendations based on qualitative discussions

**DOI:** 10.3389/falgy.2023.1052386

**Published:** 2023-02-21

**Authors:** Vibeke Backer, Lars Olaf Cardell, Lauri Lehtimäki, Sanna Toppila-Salmi, Leif Bjermer, Sietze Reitsma, Peter W. Hellings, Dan Weinfeld, Kasper Aanæs, Charlotte Suppli Ulrik, Gert-Jan Braunstahl, Bernt Bøgvald Aarli, Arild Danielsen, Hannu Kankaanranta, Sverre Steinsvåg, Claus Bachert

**Affiliations:** ^1^ Department of Otorhinolaryngology, Head and Neck Surgery and Audiology, Rigshospitalet, Copenhagen University, Copenhagen, Denmark; ^2^ Division of ENT Diseases, CLINTEC, Karolinska Institute, Stockholm, Sweden; ^3^ Department of Otorhinolaryngology, Karolinska University Hospital, Stockholm, Sweden; ^4^ Allergy Centre, Tampere University Hospital, Tampere, Finland; ^5^ Faculty of Medicine and Health Technology, Tampere University, Tampere, Finland; ^6^ Department of Allergology, Skin and Allergy Hospital, Helsinki University Hospital and University of Helsinki, Helsinki, Finland; ^7^ Department of Respiratory Medicine and Allergology, Skåne University Hospital, Lund University, Lund, Sweden; ^8^ Department of Otorhinolaryngology – Head and Neck Surgery, Amsterdam University Medical Centers, Location AMC, Amsterdam, Netherlands; ^9^ Department of Otorhinolaryngology, University Hospitals Leuven, Leuven, Belgium; ^10^ Department of Otorhinolaryngology, Upper Airways Disease Research Group, University of Ghent, Ghent, Belgium; ^11^ Asthma and Allergy Clinic Outpatient Unit (Adults), Department of Internal Medicine, South Alvsborgs Central Hospital, Boras, Sweden; ^12^ Department of Respiratory Medicine, Copenhagen University Hospital-Hvidovre, Hvidovre, Denmark; ^13^ Institute of Clinical Medicine, University of Copenhagen, Copenhagen, Denmark; ^14^ Department of Pulmonology, Franciscus Gasthuis & Vlietland, Rotterdam, Netherlands; ^15^ Department of Pulmonology, Erasmus Medical Center, Rotterdam, Netherlands; ^16^ Department of Clinical Science, University of Bergen, Bergen, Norway; ^17^ Department of Thoracic Medicine, Haukeland University Hospital, Bergen, Norway; ^18^ Department of ENT & Allergy, The Multidisciplinary Clinic “BestHelse”, Oslo, Norway; ^19^ Department of Respiratory Medicine, Seinäjoki Central Hospital, Seinäjoki, Finland; ^20^ Krefting Research Centre, Institute of Medicine, Sahlgrenska Academy, University of Gothenburg, Gothenburg, Sweden; ^21^ Department of Otolaryngology, Head and Neck Surgery, Haukeland University Hospital, Bergen and Sørlandet Sykehus, Kristiandsand, Norway; ^22^ Department of Otorhinolaryngology, International Airway Research Center, The First Affiliated Hospital of Sun Yat-sen University, Guangzhou, China

**Keywords:** asthma, nasal polyps, respiratory hypersensitivity, rhinitis, sinusitis, chronic rhinosinusitis with nasal polyps (CRSwNP), global airways disease, interdisciplinary care

## Abstract

**Background:**

Chronic rhinosinusitis with nasal polyps (CRSwNP) and asthma frequently co-exist and share pathologic features. Taking a “global” treatment approach benefits diagnosis and treatment of both, but care is often siloed by specialty: joined-up clinics are uncommon. Our objectives were to explore expert opinion to give practical suggestions to identify adults needing global airways care; enhance cross-specialty working; and widen knowledge to support diagnosis and management, integrate with existing care pathways, and supplement existing guidelines.

**Methods:**

Sixteen practicing physicians from northern Europe were invited for their national and/or international standing in treating asthma and/or chronic rhinosinusitis. Appreciative Inquiry techniques were used to guide their discussions.

**Results:**

Key themes arising were screening and referral, collaboration on management, awareness and education, and research. Provided are screening criteria and suggestions for specialist referrals, and pointers for physicians to optimize their knowledge of global airways disease. Collaborative working is underscored, and practical suggestions are given for multidisciplinary teamworking within global airways clinics. Research gaps are identified.

**Conclusion:**

This initiative provides practical suggestions for optimizing the care of adults with CRSwNP and asthma. Discussion of the role of allergy and drug exacerbations on these conditions, and care for patients with other global airways diseases were beyond scope; however, we expect some principles of our discussion will likely benefit patients with related conditions. The suggestions bridge asthma and CRSwNP management guidelines, envisioning interdisciplinary, global airway clinics relevant to various clinical settings. They highlight the value of joint screening for early recognition and referral of patients.

## 1. Introduction

Chronic upper and lower airways diseases, such as chronic rhinosinusitis (CRS) and asthma, cause substantial morbidity and mortality. Asthma accounted for some 298 disability-adjusted life-years per 100,000 people worldwide in 2017 (1), and CRS substantially impairs quality of life and carries a high socioeconomic burden (2, 3).

CRS with nasal polyposis (CRSwNP) often coexists with asthma (4, 5): asthma affects 45–49% of patients with CRSwNP (6–9) [some estimates are higher, up to 65% (10, 11)], and CRS affects up to half of those with asthma (10, 12). Both diseases associate with other airway diseases including aspirin/non-steroidal anti-inflammatory drug-exacerbated respiratory disease (AERD/N-ERD) (13, 14), eosinophilic chronic otitis media (15), and allergic rhinitis (16). Indeed, the interlinked and complex nature of airway diseases warrants a holistic management approach (17–19).

CRS is classically divided into the phenotype with nasal polyps (CRSwNP) or without (CRSsNP), and can be further subdivided by immune pathophysiology (20). CRSwNP has a prevalence of around 2.5% in Europe but is more common among those with asthma (21); polyposis also correlates with asthma severity (22, 23). Around 80% of CRSwNP cases involve intense eosinophilic airway inflammation with high immunoglobulin (Ig)-E and interleukin (IL)-5 (24, 25). However, the clinical spectrum is complex: some patients exhibit no atopic features or IgEs specific for inhaled allergens (14, 26).

Increasing understanding of the shared pathophysiological features of these conditions, their frequent co-existence, and the anatomic continuity of the upper and lower airways, has led to the concept of “global” airways diseases (19, 27–27). Mechanisms for this interaction include not just local upregulation of cytokines and adhesion molecules at mucosal sites, but systemic pathways involving the bloodstream, bone marrow and mucosa-associated lymphoid tissue (28, 29). Despite differences between compartments, the importance of treating upper and lower airway conditions together is underscored by studies showing that treating one often improves the other, and that inadequate treatment of either may exacerbate the other (19, 30).

Despite compelling evidence of the benefits a global airways approach can bring to diagnosis, treatment, and adherence, it is not yet widespread within clinical practice (31, 32). Recently, when advising the European Forum for Research and Education in Allergy and Airway Diseases (EUFOREA), European patients with long-term Type-2 airway inflammation urged better coordination between all physicians involved in treating Type-2 inflammation, whether in the upper or lower airways (33). For example, traditionally, ear, nose, and throat (ENT) physicians or rhinologists manage upper airways diseases, and pulmonologists manage lower airways diseases (33). While both specialties have specific tools and therapies to manage diseases within their area, cross-specialty collaboration and understanding, multi-disciplinary team (MDT) working, and knowledge-sharing with general practitioners (GPs), is often lacking (32, 34). This disjointed structure means current systems may hinder optimal care for patients with combined diseases (32, 34).

The advent of biologic treatment for patients with asthma in some countries (35, 36), and—more recently—CRSwNP (37), spotlights the benefits of global airways management; however, questions remain around their optimal usage (38–40). In this project, ENT physicians/rhinologists and pulmonologists from northern Europe convened to develop practical recommendations for treating adults with severe asthma and CRSwNP as a global airways disease, to try to answer the outstanding questions. Although we focused only on this specific combination, we recognize that global airways disease is a large and multi-faceted topic, and many other types—including, for example, allergic and atopic presentations—are also deserving of similar study. Our suggestions supplement existing guidelines addressing either upper or lower airways diseases, or global airways diseases together (16, 32, 41–43). This work builds on earlier consensus: in 2021, EUFOREA agreed definitions and principles of managing severe CRSwNP with biologics (43). Our work builds on this foundation by exploring how to integrate these considerations with existing care pathways, and how to improve them with cross-specialty collaboration. We explored the following hypotheses:

1. Improving global airways knowledge among specialists will facilitate timely and adequate treatment.
2. Systematic upper and lower airways assessment increases the likelihood of discovering global airways disease.
3. Collaboration between upper and lower airways physicians will improve treatment, adherence, and disease control for patients with global airways disease.

Therefore, this project aimed to provide practical suggestions for identifying and assessing upper and lower airways disease; to suggest models of collaboration between ENT physicians/rhinologists, allergologists, and pulmonologists; and to optimize treatment for patients benefiting from a global airways approach.

## 2. Materials and methods

### 2.1. Participants

The experts comprised 16 practicing physicians from Belgium, Denmark, Finland, The Netherlands, Norway, and Sweden. Each was selected for their experience in managing patients with asthma and/or CRS. Also considered was their involvement in scientific meetings, guidelines, and education, and publications records. Selection ensured a variety of care models, and equal numbers of pulmonologists and ENT physicians/rhinologists—including those also specialized in allergology—were represented.

The expert group was led by a Steering Committee of eight individuals with at least one member from each country, and co-chaired by one pulmonologist and one ENT physician. The Steering Committee guided the project's scope and provided clinical leadership.

To ensure the suggestions arising from the experts’ discussions were relevant to patients’ and primary care concerns, the all-expert meeting was joined by a practicing GP from Norway, and a patient from Finland with severe asthma and CRS.

For the purposes of this initiative, CRSwNP was defined as per the European Position Paper on *Rhinosinusitis* and Nasal Polyps (EPOS) 2020 guidelines (44).

### 2.2. Kick-off meeting, interviews and analysis of key themes

**Figure 1:**
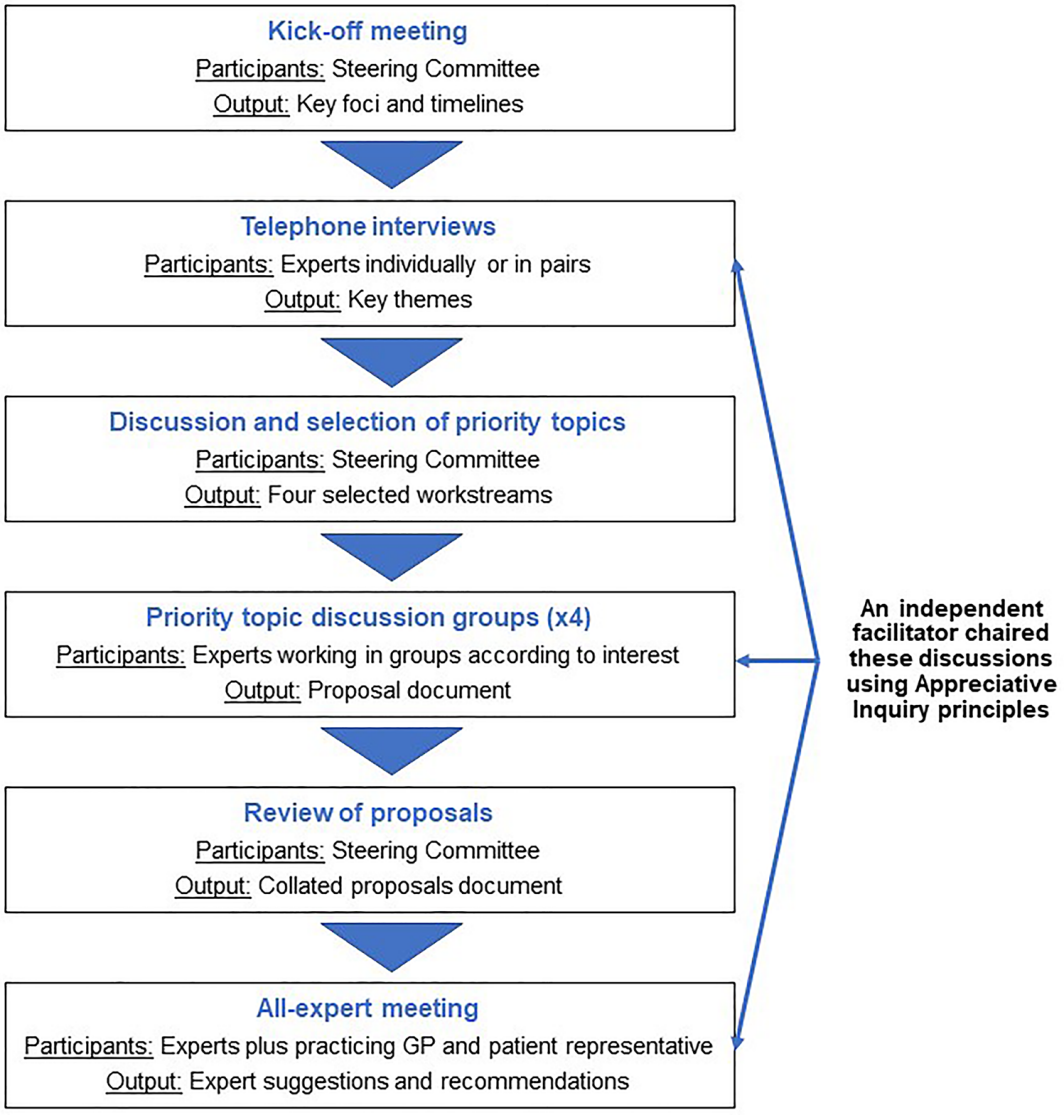
Schematic of the Appreciative Inquiry process used for this project.

This initiative was conducted between March and June 2021: its key steps are shown in Figure 1.

The Steering Committee met virtually to define the project's foci and set its timelines and milestones. This was followed by 60-minute telephone interviews with the experts, either individually or in pairs of one ENT physician/rhinologist and one pulmonologist. To avoid biasing or leading the discussions, these interviews were conducted by an independent facilitator, alongside an observer. The facilitator and observer then analyzed transcripts and contemporaneous notes to identify the key themes arising.

### 2.3. Selection of priority topics from key themes

Key themes were discussed at a Steering Committee meeting, and four priority topics among them were chosen for further exploration based on the findings. Experts were then assigned to one of four groups—one for each primary topic—according to their self-professed interests. Each group convened at least once to discuss their topic in the context of the initiative's aims, and to make suggestions. Each group submitted their proposals to the Steering Committee for review.

### 2.4. Review of proposals, and all-expert meeting

The Steering Committee used the proposals to shape an agenda for an all-expert virtual meeting. This included all participants, including the reference stakeholders. Its aims were to discuss the proposals among the wider group and to finalize the key recommendations.

### 2.5. Appreciative Inquiry approach

Appreciative Inquiry is a multi-step process that encourages participants to consolidate existing successes, not focus on problems, making positive changes more likely. Given the existence of separate recommendations for the management of asthma and CRS, Appreciative Inquiry offered a positive way to explore and unite those further, as it is an enabling process of facilitation that helps participants address evidence and its context, and to use these insights to promote evidence-based practices in healthcare (45).

## 3. Results

### 3.1. Key themes

The key ideas identified following the interviews were analyzed and arranged into themes, discussed below.

**Awareness among non-specialists of the global airways concept may be limited.**

Although “global airways” is a familiar concept to our experts, they were unsure how widely accepted it is beyond specialist settings. They suggest that applying a global airways approach in practice would involve collaboration between ENT physicians/rhinologists and pulmonologists to diagnose and manage patients in a joined-up way: from referral through diagnosis and long-term management.

**Care for those with moderate-to-severe asthma and CRSwNP is particularly siloed: adopting a “global airways” approach may greatly benefit such patients.**

The experts agreed that patients with various respiratory conditions could benefit from borderless airway management, rather than divided care from upper and lower airway specialists working separately. The experts suggested that care is particularly siloed for those with moderate-to-severe asthma and CRSwNP, and so these patients could benefit greatly from joined-up care. Nevertheless, given the cumulative burden of combined symptoms and risk of disease progression, such approaches are likely to be beneficial even to those with milder forms of disease, and for those with other airway diseases such as allergic rhinitis, AERD/N-ERD, and asthma.

**Patients are “lost in the system” because of a lack of common screening or referral approaches for patients with global airways conditions.**

Global airways disease management is not yet established in most clinical practices, and timely diagnosis is challenging. The experts report patients often becoming “lost in the system”, which was corroborated by the patient stakeholder. A lack of clear inter-specialty collaboration means that patients can bounce around within primary care and among different specialists.

**Diagnosis of comorbid asthma and CRSwNP needs improvement.**

Effective screening for combined upper and lower airways disease is vital to enable systematic diagnostic workup and referral of patients who would benefit from a global approach. Enhancing screening in primary care and in non-specialist ENT/rhinology and pulmonology settings is crucial, especially since some patients with asthma and CRSwNP respond poorly to biologics in their upper or lower airway: for them particularly, a thorough, joined-up diagnosis is important.

**Collaborative care models for patients with upper and lower respiratory diseases are heterogenous.**

To manage patients with combined disease, many experts rely on intra-specialty referrals in non-synchronous clinics. Others use informal relationships, formal agreements, or run joint ENT/pulmonology clinics focusing on specific patients. These models are determined by local healthcare settings, inter-specialist and inter-organizational relationships, local “traditions”, and established business models.

**Knowledge and culture are barriers to inter-specialty collaboration.**

Differences in clinical approaches and working cultures exist between specialties. Traditionally, ENT physicians/rhinologists have had primary interests in surgical approaches, while pulmonologists were perceived as focusing on medical approaches. This has changed as the importance of combined approaches to treating CRSw/sNP has been realized. Furthermore, in general, neither ENT physicians/rhinologists nor pulmonologists have a detailed understanding of the other's specialty, and complementary (or shared) approaches to diagnosis and management are lacking.

**Current guidelines provide only disjointed guidance for combined upper and lower airways disease.**

Existing guidelines for upper and lower airways diseases do not provide detailed, joined-up plans for holistic diagnosis and inter-specialty management. Each specialty knew the guidelines relevant to their focus, but cross-specialty awareness and understanding was low.

**The advent of biologics for CRSwNP is improving collaborative working.**

Biologics are changing the management of CRSwNP, but ENT physicians/rhinologists cannot routinely (or at all) prescribe them, owing to lacking approval or reimbursement, or organizational difficulties. The experts report the advent of biologic treatment is increasing inter-specialty engagement, perhaps because ENT physicians/rhinologists increasingly rely on pulmonology colleagues to access biologics when direct prescribing is difficult. This shift has begun to facilitate more inter-referral, communication, collaboration and MDT working.

### 3.2. Practical suggestions for global airways care

From the thematic report, the Steering Committee identified four topics for further exploration, discussed below.

#### 3.2.1. Screening and referral

All patients presenting with upper or lower respiratory symptoms in primary or secondary care should be screened for both asthma and CRS using the definitions and care pathways outlined in the Global Initiative for Asthma (GINA) (46), EPOS (21, 44), and EUFOREA (32, 43) guidelines, and according to the recommendations in Table 1.

**Table 1:** Screening for asthma and CRS.

**Screening for asthma [based on GINA 2021 (46)]:** The following features are typical of asthma and, if present, *increase* the probability that the patient has asthma: • Wheeze, shortness of breath, cough, and/or chest tightness: ○ Generally, >1 type of symptom ○ Symptoms are worse at night or in the early morning ○ Symptoms vary over time and intensity ○ Symptoms are triggered by viral infections (colds), exercise, allergen exposure, changes in weather, laughter, or irritants (e.g., car exhaust fumes, smoke) The following features *decrease* the probability that respiratory symptoms are due to asthma: • Isolated cough with no other respiratory symptoms • Chronic sputum production • Shortness of breath with dizziness, light-headedness, or peripheral tingling • Chest pain • Exercise-induced dyspnea with noisy inspiration	**Screening for CRS [based on EPOS 2020, (21) EUFOREA 2021 (32, 43)]:** CRSw/sNP is defined as the presence of ≥2 symptoms for ≥12 weeks: • Nasal blockage/obstruction/congestion • Nasal discharge (anterior/posterior nasal drip) • Facial pain/pressure • Reduction or loss of sense of smell One of which should be either nasal blockage/obstruction/congestion or nasal discharge. The combination of loss of sense of smell and bilateral nasal congestion specifically points to CRSwNP. Nasal congestion and facial pain/pressure may point to CRSsNP. Seasonal sneezing and conjunctivitis suggest allergic rhinitis.
**If asthma is suspected:** 1. Objective measurements are needed for diagnosis: a. Spirometry b. Oscillometry c. Peak-flow variability d. Diagnostic bronchial provocation test with spirometry or oscillometry (47) 2. Conduct the assessments yourself or refer, as appropriate 3. If asthma is diagnosed, assess endotype (48): a. FeNO (can be used to monitor treatment response) b. Total IgE and IgE-mediated allergies c. Blood (or sputum, if available) eosinophils	**If CRS is suspected:** 1. Assess disease history (all clinical settings including comorbidities) 2. Perform a simple nasal inspection to rule out or confirm obvious nasal polyps (all clinical settings) and then refer to specialist care 3. Perform a nasal endoscopy (specialist setting only) 4. Initiate a sinus CT scan (if nasal endoscopy is unavailable, or findings are inconclusive; specialist setting only) 5. Assess inflammation with serum IgE, and blood and tissue eosinophils
**Criteria to assess if treatment review or referral is needed in a patient with known asthma:** Asthma is well controlled, and no treatment adjustments are needed if: • Asthma symptoms are well controlled, ACT score ≥20 or ACQ score <1.2 • No more than one exacerbation per year; no hospitalizations for acute asthma • No side effects from asthma medication In other cases, adjust the treatment or refer if considered necessary.	**Criteria to assess if treatment review or referral is needed in a patient with known CRS:** Refer if: • Nasal polyposis is observed or highly suspected • Symptoms of CRSw/sNP persist with no improvement after 12 weeks of topical nasal glucocorticoids and saline irrigation
**If screening suggests the presence of moderate-to-severe asthma and CRS, consider referral to a global airways clinic. If a global airways clinic does not exist, referral should be based on the primary presenting condition, with all relevant comorbidities detailed (** **Table 2** **).**	

ACQ, asthma control questionnaire; ACT, asthma control test; CRS (w/sNP), chronic rhinosinusitis (with/without nasal polyps); CT, computed tomography; FeNO, fractional exhaled nitric oxide; GINA, Global Initiative for Asthma; IgE, immunoglobulin E.

Exact referral pathways vary among countries and clinical settings. For example, in some resource-rich countries, referrals to global airways clinics may come from allergy/immunology specialists. Therefore, the experts could not define one global pathway, although the integrated care pathway outlined in the EPOS 2020 guidelines (44) was commended. The following principles were agreed:

1. GPs, working with patients, must oversee referral and proactively integrate information between specialties, but this does not always happen in practice. Providing detailed referral letters (see Table 2)—in addition to standard medical and medication history—is desirable.
2. Specialists working in tertiary centers should collaborate, consulting together or referring patients to other specialties when appropriate. They should also proactively seek to train and educate their primary and secondary care colleagues, where appropriate, and support collaborative working.
3. Global airways clinics are recommended for complex cases. Referring directly from primary care (as well as secondary care) is appropriate.

**Table 2:** Clinical information to gather before referral.

**Both sets of information are desirable in onward referral of patients with upper and lower respiratory tract symptoms.**	
**General information, relevant to both upper and lower respiratory tract ailments** • Smoking history, occupational history, exposure to airborne irritants • Other airways diseases and allergies (symptoms and test results) • General medical comorbidities and their therapy • Medication use, including oral corticosteroids, ASA, and NSAIDs, as well as responsiveness to them
**Referral information specific to the lower respiratory tract**	**Referral information specific to the upper respiratory tract**
• Previous diagnostic testing for asthma, if any; current lung function tests • Blood eosinophil count, FeNO; spirometry results (current and previous) ○ With information on systemic GC use at the time of these tests • Previous and current asthma medication, if any; side effects • Asthma symptoms and exacerbations (in past 12 months); OCS courses; hospitalizations/ER visits • Asthma (±upper airway) symptoms triggered by ASA or NSAIDs • Biologics history; biologics eligibility assessment (i.e., absolute eosinophil count); planned biologics for asthma	• Sino-nasal symptoms (see Table 1) • Previous and current upper airways medications: ○ Nasal steroids and saline irrigation (control technique if possible) ○ Previous systemic GC for sinusitis, with details of most recent course ○ Previous nasal or sinus surgery • Assessment of adherence with, and responses to, prescribed medication • Information from anterior rhinoscopy

ASA, acetylsalicylic acid; ER, emergency room; FeNO, fractional exhaled nitric oxide; GC, glucocorticoids; NSAID, non-steroidal anti-inflammatory drug; OCS, oral corticosteroids.

#### 3.2.2. Collaboration on management

Collaboration between clinical disciplines is critical to enhance interdisciplinary knowledge and training, accelerate diagnosis, simplify referrals, minimize clinic visits, and improve patient management. Similarly, collaboration between GPs and secondary care is necessary to guide investigations, treatment, and triggers for referral.

The ideal specialist care model for patients with severe CRSwNP and asthma is *via* a dedicated MDT—a global airways clinic—including, at a minimum, a rhinologist and an asthma specialist (or allergologist) working collaboratively. A rhinologist would support diagnosis and staging of CRS and recommend optimal treatment. Asthma or allergy specialists would support asthma or allergic rhinitis diagnosis and treatment, and may provide drug challenges when assessing for N-ERD. For example, specific cyclooxygenase-2 inhibitors, if highly selective, are usually well tolerated, although this is sparsely known outside specialist settings (13, 21): collaboration would help share this knowledge. An MDT may also include other specialties, including pathologists, dermatologists, and researchers. Specialist nurses could be employed as patients’ points of contact, helping them navigate their treatment, and educating them in device usage and medication adherence.

The global airways clinic ideally should reside within a tertiary care setting, where its role should be to make overarching treatment decisions regarding types of surgery (e.g., reboot, polypectomy), and prescribe biologics. Diagnostic tests and investigations, as well as ongoing management of any chosen treatments (e.g., administering recurrent injections), should be managed in local settings, with cross-referral with global airways clinics as needed.

The precise operations of global airways clinics will vary because of local and national health service considerations and structures. However, the experts agree that, as a minimum, a global airways clinic must have the facilities and experience to perform nose and sinus surgeries, and the capacity to apply and monitor biologics use. Physicians should involve policymakers, insurance companies, patients’ boards, and local communities to organize their global airways clinic to best meet local needs.

Such structures have been implemented in The Netherlands, where collaboration between global and local clinics has underpinned their success. This global/local approach builds and concentrates in-depth knowledge in specialist centers, facilitates data collection for registries, educates clinicians working outside specialist settings, and can be more convenient for patients. Global airways clinics also exist in some Danish hospitals, in which nasal polyps and lung function are assessed together. This approach increases staffing and associated costs, but its key advantage is that different specialists collaborate to evaluate patients once, then diagnose and treat individually. Nurses follow up regularly with patients.

Table 3 describes the experts’ specific advice for healthcare settings considering establishing a global airways clinic.

**Table 3:** Practical advice for establishing a global airways clinic.

Step	Suggestion
Where to start:	Start with small-scale collaboration between interested specialists, ideally in the same hospital, to review difficult cases of patients with moderate-to-severe asthma and CRSwNP.
How to meet:	Ensure dedicated, regular times are scheduled when a consistent team can attend.
How to collaborate:	Develop a consistent and coordinated approach to data management using common tools, files, and databases.
What tools to use:	Use existing electronic tools to support real-time data collection, help patients track their symptoms, and enable clinicians access to the latest information.

CRSwNP, chronic rhinosinusitis with nasal polyps. Although the focus of this project was asthma and CRSwNP, the recommendations here may be applicable for other global airway diseases, e.g., allergy and rhinitis (CRS).

#### 3.2.3. Awareness and education

**Figure 2:**
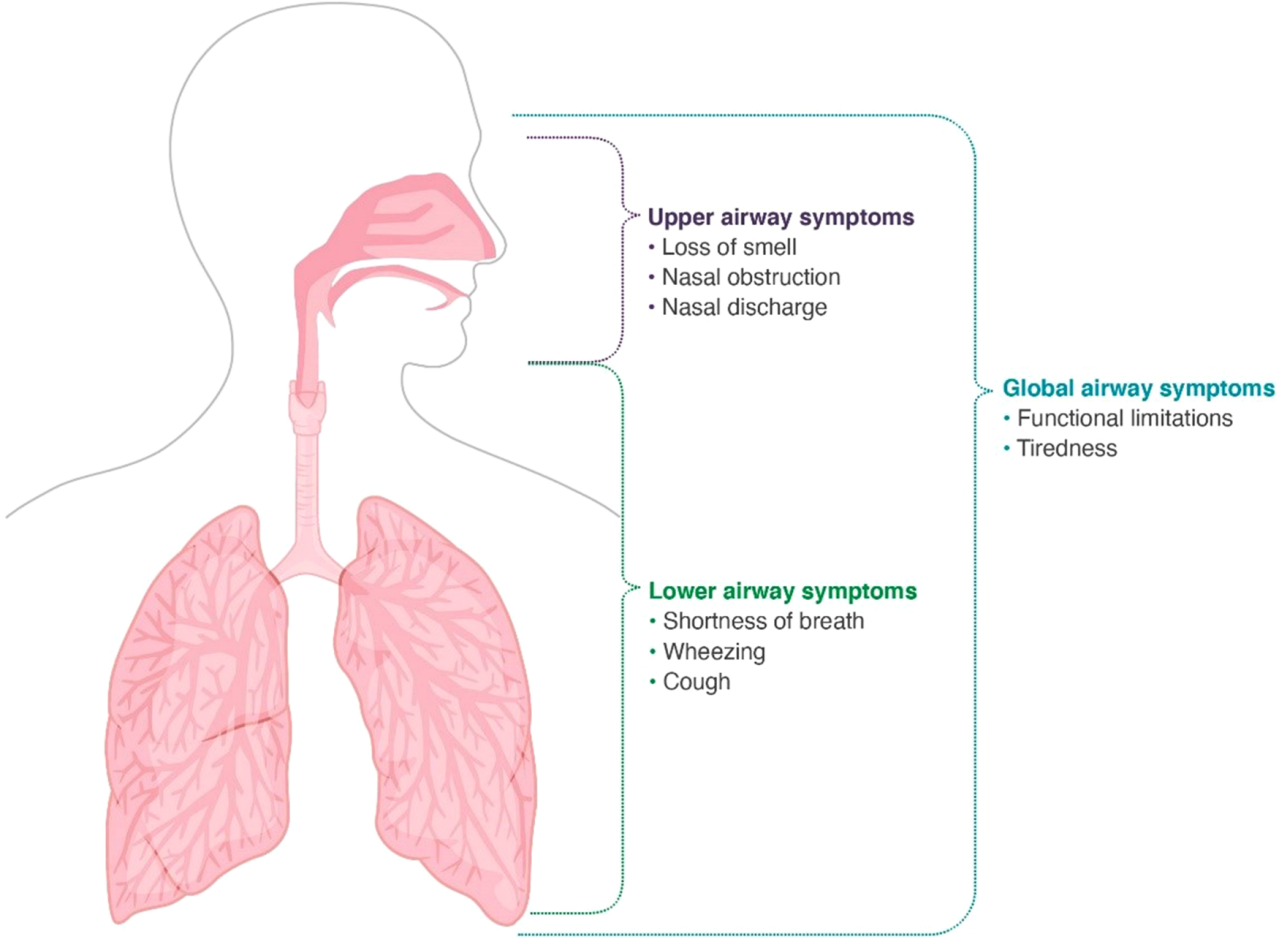
Key differentiating symptoms of combined severe asthma and chronic rhinosinusitis with nasal polyps. Additional symptoms associated with other airways conditions may also be present including for allergic rhinitis: runny nose, itchy nose, eyes and ears, sneezy, nasal discharge, seasonal variation; for chronic rhinosinusitis: loss of smell, nasal closure/obstruction, facial headache, nasal discharge; and additionally for asthma: chest tightness, night time symptoms, exercise-induced symptoms.

Improving diagnosis, referral, and treatment depends on raising awareness of global airways, as well as ensuring patients, GPs, and specialists can confidently recognize and treat upper and lower airways symptoms. Recognition of the key differentiating symptoms of combined severe asthma and CRSwNP (Figure 2) is vital, as is awareness of guideline-recommended treatment paradigms (Table 4).

**Table 4:** Overview of the treatment paradigms for moderate-to-severe asthma and CRSwNP.

**Based on GINA 2021 (46)** **Adults and adolescents aged 12+ years with severe asthma**	**Based on EPOS/EUFOREA 2020/2021 (32, 44)** **Uncontrolled severe Type-2 CRSwNP**
**Initial treatment of a severely uncontrolled asthma, or an acute exacerbation:** Start regular controller treatment with high-dose ICS, or medium-dose ICS-LABA. A short course of OCS may also be needed. Review patient's response after 2–3 months, or earlier depending on clinical urgency. If symptoms occur daily for months, or if the patient wakes up with asthma more than once a week, or has low lung function. • **Preferred controller:** Medium-dose ICS-LABA ○ Other controller options: high-dose ICS, add-on tiotropium/LTRA ○ Consider adding HDM (or other allergens) SLIT for sensitized and exposed patients with allergic rhinitis and FEV_1_ >70% predicted • **Preferred reliever:** As-needed low-dose ICS-formoterol ○ Other reliever option: As-needed SABA. In patients not on regular ICS (GINA step 1, mild asthma), ICS should be taken together with the as-needed SABA Can escalate further to include: • Add-on LAMA, possibly as a triple treatment (ICS/LABA/LAMA) • High-dose ICS-LABA • Referral for phenotypic assessment ± add-on therapy, e.g., anti-IgE, anti-IL-5/5R, anti-IL-4R • Macrolides for long-term treatment Step down treatment once good control has been maintained for 3 months. **Note:** While CRS can contribute to respiratory symptoms, e.g., chronic cough, its treatment in patients with asthma should be targeted at the symptoms of rhinosinusitis rather than to improve asthma control.	**Overview of therapeutic strategy for uncontrolled severe Type-2 CRSwNP:** The cornerstone of CRSwNP and asthma management consists of anti-inflammatory treatment with local corticosteroids. When this is insufficient, short courses of OCS may be used (usually 30–60 mg for 14 days, sometimes reducing over time). **Appropriate medical treatment:** • Nasal corticosteroid (drops/spray/rinses) • Saline rinses • Education on technique/compliance • Consider OCS and endoscopic sinus surgery If insufficient improvement in 6–12 weeks, consider: • Extended/Reboot surgery in qualified centers (49) • Biologics, eventually in combination with surgery • In rare cases, ASA therapy after desensitization [if N-ERD (50)] Consider biologics for patients with CRS and bilateral nasal polyps and: • Evidence of Type-2 inflammation • Need for OCS (≥2 courses in the last year) or surgery • Significantly impaired quality of life • Significant loss of sense of smell • Comorbid asthma

ASA, acetylsalicylic acid; CRS, chronic rhinosinusitis; CRSwNP, chronic rhinosinusitis with nasal polyps; EPOS, European Position Paper on Rhinosinusitis and Nasal Polyps; EUFOREA, European Forum for Research and Education in Allergy and Airway Diseases; FEV_1_, forced expiratory volume during 1 s; GINA, Global Initiative for Asthma; HDM SLIT, house dust mite sublingual immunotherapy; ICS, inhaled corticosteroids; Ig, immunoglobulin; IL, interleukin; LABA, long-acting β2-agonist; LAMA, long-acting muscarinic antagonist; LTRA, leukotriene receptor antagonist; N-ERD, non-steroidal anti-inflammatory drug-exacerbated respiratory disease; OCS, oral corticosteroids; R, receptor; SABA, short-acting β2-agonist.

Cross-specialty education would accelerate patients’ identification and referral, and prevent patients from becoming “lost in the system” without holistic care from any provider. Education should be tailored to each audience. Respiratory nurses’ roles could be expanded to help educate primary care practitioners, who should themselves focus on screening for comorbid diseases, investigations helpful for secondary care, and referrals. In secondary care, education should be delivered jointly for involved specialties to develop a common understanding of global airways disease and its management.

#### 3.2.4. Research

Collaborative care of patients with global airways disease is advocated in guidelines, and anecdotal examples demonstrate its value, but further consolidating research is needed. Evaluation tools to stage and characterize patients with combined disease are desirable, and could be used to guide their management. The experts identified five topics deserving of priority research:

1. If a composite tool existed to aid screening and referral to secondary or tertiary care, which symptoms should, and could, it measure?
2. Would early intervention in patients with combined upper and lower airways disease reduce their total inflammatory burden? Would this reduce their future risk of deterioration and/or systemic consequences (e.g., increased total disease burden including comorbid conditions)? How cost-effective is this approach?
3. What measurable impact does collaborative management have on patients? What evidence already exists? Which endpoints should we measure to assess its impact?
4. What is the impact of treating a patient’s asthma on their nasal polyposis, and vice versa? More evidence is needed for individual treatments and disease phenotypes.
5. What is the role of endotyping and/or phenotyping in diagnosing and managing combined airways disease?

## 4. Discussion

This project gathered specialists in managing patients with some of the most intractable upper and lower respiratory diseases. This group considered how to practically implement global airways screening, assessment, diagnosis, and management across disciplines and clinical settings to enhance patients’ care.

The experts suggest screening criteria for asthma and CRS, suggesting what to do if either is suspected, what information to gather, and when to refer to specialist care. They underscore the importance of collaborative working between all physicians involved in these patients’ care, primarily *via* a dedicated MDT working in a global airways clinic, ideally in a tertiary setting. The experts advise how to establish such a clinic within existing frameworks. The experts give care providers ways to optimize their awareness of symptoms and guideline-recommended treatment paradigms. Lastly, the experts identify research questions to optimize the care of patients with global airways disease in future.

We focused on patients with moderate-to-severe asthma and moderate-to-severe CRSwNP, for whom treatment paradigms are changing given the advent of biologic therapies, and for whom existing care is particularly siloed (51). Biologics now offer the possibility of combining medical and surgical solutions (32, 43, 44), and although this shift is encouraging, the absence of multi-disciplinary working is evident (43). However, we recognize that asthma and CRSwNP is just one of many manifestations of global airways disease that could benefit from better joined-up working, and hope that our principles will have broader application to other indications.

The suggestions arising from this initiative supplement, but do not replace, guidelines for asthma (41, 42, 46) and CRS (21, 44), by augmenting them with practical advice for clinicians wishing to collaborate and implement global airways approaches in their practice.

The difficulties arising from managing respiratory tract conditions as distinct—often siloed—specialties were a recurring theme in this initiative, and this echoes concerns repeatedly raised by patients in a recent EUFOREA study (52). Vital to the success of our initiative was to build on separate established traditions and best practices in the management of upper and lower airways diseases. Using Appreciative Inquiry, we explored how existing successes could be broadened and united. In healthcare, Appreciative Inquiry helps people consider evidence, its context and its application (45). Appreciative Inquiries typically involve large groups working face-to-face over several days; however, coronavirus restrictions necessitated a virtual approach. This may have slowed the project's momentum, but nevertheless enabled deep discussion and reflection.

Our vision for collaborative care of patients with moderate-to-severe global airways disease has three parts: dedicated teams of specialists collaboratively manage the most severely affected patients in global airways clinics; GPs and non-specialists understand global airways disease and can quickly screen and appropriately refer patients; and informed patients can identify their symptoms as global airways disease, advocate for themselves, and self-manage their own care. The gold standard recommended here is a multi-disciplinary approach, but we accept this may be difficult to achieve in all health settings, although digital solutions may make this more accessible in future.

The wider adoption of telemedicine during the COVID-19 pandemic, especially in secondary care (53), may now enable the use of virtual multi-disciplinary consultations. This could aid initial patient assessment and support appropriate referral to tertiary settings, potentially streamlining the process and expanding patient access especially in areas where health resources are limited.

At the time of our initiative, no composite screening tools for assessing upper and lower respiratory tract signs had been developed. In the absence of such a tool, we highlighted the concurrent use of existing screening approaches for asthma and CRS as a pragmatic alternative, building on those already validated and available. However, success would depend on clinicians having a greater understanding of global airways disease and its impact on referral pathways. While not available at the time our initiative was done, the STARR-15 tool now offers the first global airways questionnaire to be used when examining patients with upper and lower airways symptoms, such as allergic rhinitis, CRS and asthma (54). While pending validation, we hope that this and future tools may help improve the diagnosis of patients with global airways diseases, which is among our key recommendations.

Integrated care is common when treating patients with comorbid conditions. For example, Allergic Rhinitis and its Impact on Asthma (ARIA) advocates this approach for managing allergic rhinitis and asthma as one disease (41), and this is desired by patients with various global airways diseases (51, 52). Again, these pathways need to be identified locally and supported by targeted education for clinicians in primary care and non-specialist settings.

Shared decision-making and patient empowerment are essential (43). We advocate for a more symptoms-based approach to recognizing global airways disease, and suggest focusing on the symptoms most indicative of Type-2 disease.

This project's limitations mainly relate to the breadth of expert and non-expert involvement. A small number of experts were involved, albeit selected for their understanding of their countries’ health systems. Most were ENT/rhinology consultants or pulmonologists (with some specializing in asthma and allergology), and most work in university or tertiary care settings. Although representative of the specialists encountering the most severe cases, we recognize we may have overlooked the nuanced viewpoints from other disciplines (such as pathology or pharmacy). In addition, ENT physicians/rhinologists and pulmonologists working outside specialist settings were not included. Overall, expert selection ensured the group was weighted for experts familiar with the patients in question, and with experience implementing collaborative practices.

Patients and primary care practitioners were under-represented in our expert group, and no specialist nurses were involved: in retrospect, we recognize the group would have benefited from earlier and more extensive input from these stakeholders. However, the consensus underscores the need to engage these groups locally, to build and embed referral practices, and to improve communication and symptom recognition.

The recommendations herein are based on clinical practice in northern European countries, while considering their heterogeneity. We suggest they may therefore have wider relevance to similar high-income health settings. We hope these first steps to collaborative clinical practice will be developed further, perhaps by a collaborative working group spanning the organizations focused on global airways disease. In parallel, further evidence is needed to understand the value of different treatment models, as is a validated, composite tool to identify patients with combined disease burden. We suggest that, in future, such topics may lend themselves well to more formalized consensus recommendations using Delphi or similar methods.

In conclusion, this initiative gives practical suggestions for better identifying patients who would benefit from a global airways approach, enhancing referral pathways and promoting collaborative cross-specialty working and education. We have specifically focused on patients with CRSwNP and comorbid severe asthma, although these principles will likely benefit patients with more wide-ranging, difficult-to-treat, or even less-severe indications that nevertheless need better recognition. We hope this initiative stimulates reflection, prompts cross-functional discussion, and renews focus on improving the care of patients with global airways disease.

## Data Availability

The original contributions presented in the study are included in the article, further inquiries can be directed to the corresponding author.
